# Scaled in Cartesian Coordinates Ab Initio Molecular Force Fields of DNA Bases: Application to Canonical Pairs

**DOI:** 10.3390/molecules27020427

**Published:** 2022-01-10

**Authors:** Igor Kochikov, Anna Stepanova, Gulnara Kuramshina

**Affiliations:** 1 Research Computing Centre, Moscow State University, 119991 Moscow, Russia; igor@kochikov.ru; 2 Chemistry Department, Moscow State University, 119991 Moscow, Russia; stepanna@hotmail.ru

**Keywords:** molecular force field, cartesian coordinates, scaling factors, NBO theory, DNA bases, DNA canonical base pairs

## Abstract

The model of *Regularized Quantum Mechanical Force Field* (RQMFF) was applied to the joint treatment of ab initio and experimental vibrational data of the four primary nucleobases using a new algorithm based on the scaling procedure in Cartesian coordinates. The matrix of scaling factors in Cartesian coordinates for the considered molecules includes diagonal elements for all atoms of the molecule and off-diagonal elements for bonded atoms and for some non-bonded atoms (1–3 and some 1–4 interactions). The choice of the model is based on the results of the second-order perturbation analysis of the Fock matrix for uncoupled interactions using the *Natural Bond Orbital* (NBO) analysis. The scaling factors obtained within this model as a result of solving the inverse problem (regularized Cartesian scale factors) of adenine, cytosine, guanine, and thymine molecules were used to correct the Hessians of the canonical base pairs: adenine–thymine and cytosine–guanine. The proposed procedure is based on the block structure of the scaling matrix for molecular entities with non-covalent interactions, as in the case of DNA base pairs. It allows avoiding introducing internal coordinates (or coordinates of symmetry, local symmetry, etc.) when scaling the force field of a compound of a complex structure with non-covalent H-bonds.

## 1. Introduction. Inverse Problems of Vibrational Spectroscopy

Vibrational spectroscopy is a very important source about the structure of molecules, in particular, in different states of aggregation, including the presence of intra- and intermolecular interactions. Currently, there is a large number of available infrared and Raman spectra, measured with a sufficiently high accuracy, which, as a rule, are supplemented by quantum mechanical calculations at the modern level of theory.

In this article, we consider new possibilities offered by our earlier proposed method for solving the inverse vibrational problem (finding the matrix of force constants of a molecule) by calculating the scale factors directly in Cartesian coordinates [1], namely, the application of this approach to correcting the theoretical frequencies of molecular associates with non-covalent interactions. Generally, the empirical force field is defined as a set of parameters of an individual molecule (or its associates of the finite dimension), which are determined from experimental data on molecular geometry and sets of vibrational frequencies of an individual molecule by solving the so-called inverse vibrational problem. These “small” force fields of separate molecules are widely used in modern computational chemistry as a part of the extended biomolecular force fields for simulating bulky biological systems by MD or MM methods.

The concept of molecular force field arises within both classical and quantum mechanics when a molecule is considered as a mechanical system of nuclei, while all interactions of electrons are included in the effective potential function *U*(*q*_1_, …, *q_n_*), where *q*_1_, …, *q_n_* denote *n* = 3*N*–6 generalized coordinates of *N* atomic nuclei of the molecule. The minimum potential function (with respect to nuclei coordinates) defines the equilibrium geometry of a molecule. The second derivatives of the potential with respect to nuclei coordinates in equilibrium:

$$f_{ij}=\frac{\partial^{2}U}{\partial q_{i}\;\partial q_{j}}\;\left|{}_{eq}\right.\;\;(i,j=1,\ldots n)$$

constitute a positive defined force constant matrix *F* determining all the molecular characteristics related to small vibrations. The vibrational frequencies (obtained from IR and Raman spectra) are the main type of experimental information on molecular vibrations. They are connected with the force constant matrix by the eigenvalue equation:

(1) GFL=LΛ

The parameters of the empirical force field are determined by processing the data of experimental infrared and Raman spectra. The so-called *inverse vibrational problem* of determining the parameters of the molecular force field (matrix of force constants, *F*) from the given experimental data (vibrational frequencies, isotope frequency shifts, Coriolis constants, centrifugal distortion constants, etc.) is formulated [1] in the form of a nonlinear operator equation in finite-dimensional spaces:

(2) $$A_{h}F=\Lambda_{\delta }$$

where *F* ∈ *Z* ∈ *R^n^*^(*n*+1)/2^ (*Z* is a set of possible solutions) is the unknown force constant matrix (real and symmetric), Λ ∈ *R^m^* represents the set of available experimental data (vibrational frequencies, etc.) determined within *δ* error level: $\left\|\Lambda -\Lambda_{\delta }\right\|\leq \delta$. *A* is a non-linear operator which maps matrix *F* on Λ, *h* is an estimate of the operator *A* uncertainty. The accumulation of data on force constants is necessary for predicting the spectra and other properties of compounds not yet investigated and for the development of physical models in the theory of molecular structure.

This mathematical problem of calculating molecular force fields within the general approximation of small vibrations (harmonic model) belongs to the class of mathematical non-linear ill-posed problems [2]. Ill-posedness means that the problem does not satisfy any of the three well-posedness conditions (the existence of a solution, its uniqueness, and stability with respect to perturbations in the input data) [3]. In most cases, the main difficulty in solving such problems is connected with the non-uniqueness of the solution. Indeed, for any molecular structure (except for diatomic molecules), there may exist an infinite number of force field matrices that result in the same set of vibrational frequencies.

At the same time, the increasing possibilities and a rather good accuracy of the modern-state calculations within ab initio and density functional theory approximations have opened up real ways to obtain the force field parameters from high-level computations of structural units, which can be identified with the help of the modern structural methods. Among these units, there are various molecular clusters/associates that are formed due to non-covalent interactions such as H-bonds or stackings. These interactions can be identified from corresponding infrared and Raman spectra, which also contain information about the conformational composition of the compound. The force fields of such molecular systems with non-covalent bonds between separate units should include an intramolecular part responsible for the corresponding vibrations. Quantum-mechanical calculations of molecular clusters allow predicting both the spectral part responsible for intermolecular vibrations (vibrational IR and Raman spectra) of individual fragments and the spectral part responsible for intramolecular vibrations involved in large-amplitude motions associated with conformational changes in biomolecules.

The solving of ill-posed problems can only be realized with the use of stable numerical methods. For solving the inverse vibrational problem, there have been proposed methods based on the theory of regularization of non-linear ill-posed problems [1,2]. The main idea of this theory is as follows: to solve an ill-posed inverse problem, it is necessary to formulate an algorithm and an optimization procedure that provide a unique solution to a mathematical problem. Such a solution can be obtained using a stable numerical algorithm with the inclusion of mathematically formulated additional criteria for choosing a single solution with given properties [1,2]. This approach allows searching for the so-called normal pseudosolution of Equation (2). Such a solution is determined as an optimized matrix of force constants closest in the chosen Euclidean norm to the a priori given matrix *F*^0^. The solution must satisfy the set of constraints *D* and reproduce the experimental data Λ*_δ_* within a given error level. *D* is a given set of a priori constraints (supposed to be closed), which describe various types of constraints on the force constant values [3,4].

Within the framework of the regularization theory, such stable solution (*F^α^*) can be obtained as an extreme of the Tikhonov’s functional [1,2,3,4]:

(3) $$M^{\alpha }[F]={\left\|A_{h}F-\Lambda_{\delta }\right\|}^{2}+\alpha {\left\|F-F^{0}\right\|}^{2}$$

on the set *D* where *F*^0^ is some a priori chosen stabilizing matrix. The existence of an extreme *F^α^* is proved in [3,5]. To obtain a stable solution, the regularization parameter *α* should be chosen in accordance with the errors (*h*, *δ*) in geometry and experimental frequencies, respectively. The result of minimization is the matrix *F^α^* closest to the given matrix *F*^0^ and compatible with the experimental data within the specified error level.

In 1994, in collaboration with F. Weinhold, the group of scientists from Moscow State University proposed using stable numerical algorithms based on Tikhonov’s regularization method for joint treatment of ab initio and experimental data in molecular force field calculations [6]. It was suggested to ‘‘regularize’’ and stabilize quantum-mechanical force fields by means of finding the so-called normal solution (pseudo-solution) of the inverse vibrational problem. In this model, the stabilizing matrix *F*^0^ is chosen from quantum mechanical calculations, and the resulting solution will be the matrix *F^α^* which is the closest in the Euclidean norm to the given ab initio *F*^0^. The optimized solution is the so-called *Regularized Quantum Mechanical Force Field* (RQMFF) [1,6]. The force constant matrix *F^α^* obtained in this way reproduces the experimental frequencies with given accuracy and is the closest (in the sense of the Euclidian norm) to the specified ab initio matrix *F*^0^ describing the intramolecular interactions. The proposed procedure allows the use of any system of generalized coordinates, including redundant systems of internal or symmetry coordinates [1], which simplifies the transferability of force constants between related molecules. Using regularizing algorithms to refine ab initio force fields, it is possible to obtain solutions to the inverse vibrational problem that retain significant features of the ab initio force constant matrix. In particular, it allows to keep the potential energy distribution (PED) or composition of normal-mode eigenvectors, thereby providing accurate use of information obtained by powerful ab initio methods, transfer, and comparison of force constants in a series of related molecules. The proposed RQMFF approach was successfully used in our joint studies with F. Weinhold [7,8,9,10] carried out for a series of substituted alkanes with the goal of determining the regularities in molecular parameters upon fluoro–chloro substitution.

Later, this approach to solving the inverse vibrational problem was extended for the very popular Pulay model of scaled force constant matrix (expressed in internal or symmetry/local symmetry coordinates) [11,12,13]. The corresponding regularizing algorithms provide the matrix *F^α^* with the following properties: the solution is closest by norm to the QM matrix *F*^0^, or the scale matrix *B* is closest to the unit matrix [1,14].

## 2. Results

### 2.1. Scaling of Molecular Force Fields in Cartesian Coordinates

Quantum chemical calculations provide the Hessian matrix of the second derivatives of the energy with respect to atomic coordinates. As a rule, the interpretation of theoretical results is carried out in some selected system of internal coordinates related to the geometric parameters of the molecule (bond stretchings, bond angles, dihedral angles, etc.). In the case of large molecular systems, the introduction of a complete system of internal coordinates is the most tedious and time-consuming procedure. Moreover, if we consider the intramolecular part of the force field for macroscopic systems expressed in terms of force constants corresponding to molecular internal coordinates (bond lengths, valence, and dihedral angles), then we encounter a significant problem related to the invariance of individual internal force constants with respect to the chosen set of coordinates: the force constants of the intramolecular force field are not invariant with respect to the choice of internal coordinates.

In the case of large molecular systems, the introduction of a complete system of internal coordinates is the most tedious and time-consuming procedure. Moreover, if we consider the intramolecular part of the force field for macroscopic systems, expressed in terms of the internal coordinates of molecules (bond lengths, bond, and dihedral angles), then we are faced with a significant problem. It consists in the fact that the force constants of intramolecular force fields are not invariant with respect to the choice of internal coordinates.

The simplicity of the scaling procedure has made it extremely popular. It has been shown that the scale factors of many molecular fragments (within a given level of quantum-mechanical method) are approximately constant over a wide range of similar molecules. Initially, the scaling procedure was suggested [11,12,13] for the force fields defined in an internal or symmetry (local symmetry) coordinate system. A similar approach for scaling molecular force fields in internal/symmetry/local symmetry coordinates was implemented in our software [1,14,15] developed for solving direct and inverse vibrational problems.

Internal coordinates (including interatomic distances, bond angles, dihedral angles, etc.) have a lot of advantages and are closely related to the assumptions of the classical theory of structure. They are commonly used to characterize molecular geometry and to define energy terms in valence force-field models. These coordinates provide a language that can be easily used in many applications. The force constant matrix expressed in terms of internal coordinates has a clear advantage. However, unfortunately, the total number of force constants in such a matrix is equal to *n*(*n* + 1)/2, where *n* = 3*N* − 6 (*N* is the number of atoms in a molecule). For example, in the case of adenine–thymine base pair, the total number of atoms *N* is equal to 30. To describe this system in internal coordinates, it is necessary to use at least 84 internal coordinates. The number of the force constants (expressed in independent internal coordinates) is 3550. Even if some of the force constants in internal coordinates are chosen equal to zero, the total number of force constants remains quite large.

Regularizing algorithms allow using any system of generalized coordinates, including redundant systems of internal coordinates, which greatly facilitates the transfer and comparison of force constants between related molecules. The theoretical basis and practical aspects of using redundant coordinates in molecular force fields calculations were previously discussed [1].

To avoid the problems arising in defining the set of internal coordinates, especially in the case of large molecules, we have proposed a procedure for scaling ab initio force field matrix in Cartesian coordinates [16]. This problem is not trivial because the scaling matrix for the Hessian in Cartesian coordinates cannot be chosen as diagonal. However, it is possible to formulate certain conditions allowing to find appropriate scale factors, which are discussed below. The model based on scaling in Cartesian coordinates can also be used in symmetry coordinates and significantly reduce the dimension of the mathematical problem [16,17], so it has an obvious advantage in the case of rather bulky molecules, such as a smaller dimension of coordinate space and, accordingly, a smaller number of optimized parameters. One of the main advantages of this approach is that it avoids introducing internal coordinates in the process of scaling and transferring force fields. Numerical details of this procedure and some details of the use of symmetry in this approach, as well as examples of determining the scale factors in Cartesian coordinates of various organic molecules, were presented in our previous publications [16,17,18].

The procedure of scaling the quantum-chemical force matrix *F*^0^ in internal coordinates is defined as:

*F = BF^0^B* (4)

where *B* is a diagonal matrix composed of scale factors.

Let *A*(*F*) be an operator that puts into correspondence to a symmetric positively defined force constant matrix *F* a vector consisting of vibrational frequencies; then the problem of finding the scale factors can be formulated as a non-linear operator equation:

*Q(B)* = Λ (5)

where *Q*(*B*) = *A*(*BF*^0^*B*), and *Q*(*B*) is a continuous finite-dimensional operator.

The solution of the inverse problem in Cartesian coordinates has specific features that are determined by the constraints imposed on the force constant matrices: namely, the molecular potential energy must be independent of translations or rotations of a molecule as a whole. An explicit form of these constraints was presented in our previous papers [16,17,18]. These constraints lead to a decrease in the matrix rank to *3N* − 6, where *N* is the number of atoms. Obviously, these constraints must be maintained while scaling the Cartesian matrix.

Fitting molecular force fields in Cartesian coordinates reduces the difficulties associated with the choice of internal coordinates in complex molecules and is practically useful in the case of large biological molecules, associates, polymers, etc., including hundreds of atoms, for which only moderately accurate quantum chemistry methods can be applied. To calculate the scale factors in Cartesian coordinates, a special routine of the SPECTRUM2 software package was used [16].

In the case of scaling in Cartesian coordinates, the scaling procedure can also be formulated in the form of Equation (4). However, it was shown [16,17] that the scale factor matrix *B* cannot be chosen diagonal due to the requirement that the matrix *BF*^0^*B* is independent of the translations and rotations of the molecule as a whole. This requirement imposes certain constraints on the elements of matrix *B*. As a result, the problem of finding the scale factors is formulated as Equations (4) and (5), where *B* is a symmetric matrix, *B* ∈ *D* where *D* is a set of the mentioned constraints. In addition, set *D* may include symmetry constraints.

Fitting molecular force fields in Cartesian coordinates reduces the difficulties related to the choice of internal coordinates in complex molecules and is practically useful in the case of large biological molecules, associates, polymers, etc., including hundreds of atoms. Numerical methods for solving an inverse vibrational problem of the form (4–5) were formulated and applied in [14,15]. The solution to the inverse problem (a set of scale factors) is found by minimizing the functional:

(6) $$M^{\alpha }[B]={\left\|Q_{h}(B)-\Lambda_{\delta }\right\|}^{2}+\alpha {\left\|B-E\right\|}^{2}$$

on the set *B* ∈ *D* with the proper choice of the regularization parameter *α*. Here, *Q_h_* and Λ*_δ_* represent approximations of the operator *Q* and a set of experimental vibrational frequencies Λ, respectively [16,17,18].

In this paper, we present the results of applying these algorithms for fitting the molecular force fields in Cartesian coordinates for primary nucleobases (adenine, cytosine, guanine, and thymine) and subsequent use of these scale factors to correct the force constant matrices of canonical pairs in Cartesian coordinates.

### 2.2. Application of Second-Order Perturbation Theory Analysis to the Fock Matrix in NBO Basis for DNA Bases and Base Pairs at the B3LYP/6-31G* Level of Theory

The model of scaling in Cartesian possesses many advantages in over scaling in other generalized coordinates. This approach requires careful analysis of the scaling matrix structure, especially in cyclic molecules, for which it can be difficult to estimate all-important pairwise interactions of atoms. One of the very attractive possibilities for predicting the possible structure of the scaling matrix in complex cyclic systems is the analysis of pairwise interactions of atoms in the framework of the theory of Natural Bond Orbitals (NBO) proposed in the works of Frank Weinhold [19,20,21]. This theory allows to obtain information on charge transfer or conjugative interactions in molecular systems and can be characterized as a very reliable, sensitive, and one of the most efficient theoretical tools for the analysis of intra- and inter-molecular interactions by using calculated data about the interaction of filled and virtual orbitals.

In this work, quantum mechanical calculations and NBO analysis of considered systems were carried out at the B3LYP/6-31G* level of theory. The 6–31G(d) basis set was chosen as one of the simplest polarized double-zeta basis sets, which is widely used in quantum mechanical calculations of bulky biological molecules. Some results of applying the second-order perturbation theory analysis to NBO basis for DNA base molecules at the B3LYP/6-31G level of theory can be found in Table 1 and Table 2, compared to the similar calculations for base pairs. 

The visualization of optimized molecular structures has been made using Chemcraft (version 1.8) software [22]. The results of NBO analysis demonstrate the presence of significant hyperconjugative interactions between lone pairs of nitrogen and oxygen atoms with antibonding orbitals (σ*) of some skeletal bonds both in DNA base molecules and in their pairs. Therefore, it is necessary to include certain cross-sectional terms in the scaling matrix.

### 2.3. Scaling of Molecular Force Fields in Cartesian Coordinates

#### 2.3.1. Computational Details

DFT calculations of four DNA bases (adenine, thymine, cytosine, and guanine) and their pairs (adenine–thymine and cytosine–guanine) were performed using the GAUSSIAN 09 software (Revision D.01) [23]. Fully optimized geometries, analytical force constants, and harmonic vibrational frequencies of all molecular structures were calculated at the B3LYP/6-31G* level of theory [24,25,26]. Potential surface minima were found by relaxing geometric parameters using standard optimization methods. Inverse scaling problems were solved for all bases, and the resulting sets of scale factors for DNA bases were found as the extremes of functional (6) for each base. The calculation of scale factors was carried out using a special routine of the SPECTRUM software package [1], which was equipped with additional options for applying to Cartesian coordinates. All the considered inverse problems were solved for single parent molecules without the inclusion of experimental data on isotopic species to avoid the incompatibility of inverse problems in the framework of the harmonic model.

#### 2.3.2. The Short Protocol of Calculations

- Quantum mechanical calculations of optimized geometries and harmonic force fields of adenine, cytosine, guanine, and thymine, as well as of the two pairs adenine–thymine and cytosine–guanine at several levels of theory (B3LYP/6-31G*, B3LYP/6-311++G**, PBEPBE/DGDZVP, BVP86/TZVP).

The choice of these levels of quantum mechanical calculations is explained by their popularity in quantum mechanical calculations of organic molecules and, especially, biological molecules. In this paper, we present results obtained for the B3LYP/6-31G* level since this level of calculations can be applied to larger biologically important molecules.

- Analysis of various matrix scaling models in Cartesian coordinates based on the results obtained in the framework of the second-order perturbation theory analysis of the Fock matrix in the NBO basis for the DNA bases under consideration.
- Solving inverse problems and determining the scale factors sets in Cartesian coordinates for four DNA bases at the B3LYP/6-31G* level of theory.
- Solving inverse problems with a variation of possible sets of scale factors.
- Finally, scaling the force constant matrices for DNA pairs. Scale matrix for each pair was composed in block-diagonal form. Comparison to available experimental spectra if appropriate.

The calculated scaling matrices in Cartesian coordinates (B3LYP/6-31G*) of DNA bases are presented in Supplementary Materials Tables S1–S4, comparison of the fitted and observed frequencies are presented in Supplementary Materials Tables S5–S8.

#### 2.3.3. Results of Quantum Mechanical Calculations and Fitting Scale Factors for DNA Bases

All four DNA bases were processed similarly to obtain scale factor matrices *B*. Below, we demonstrate the results of calculations for the adenine molecule obtained at the B3LYP /6-31G* level of theory. 

**Figure 1:**
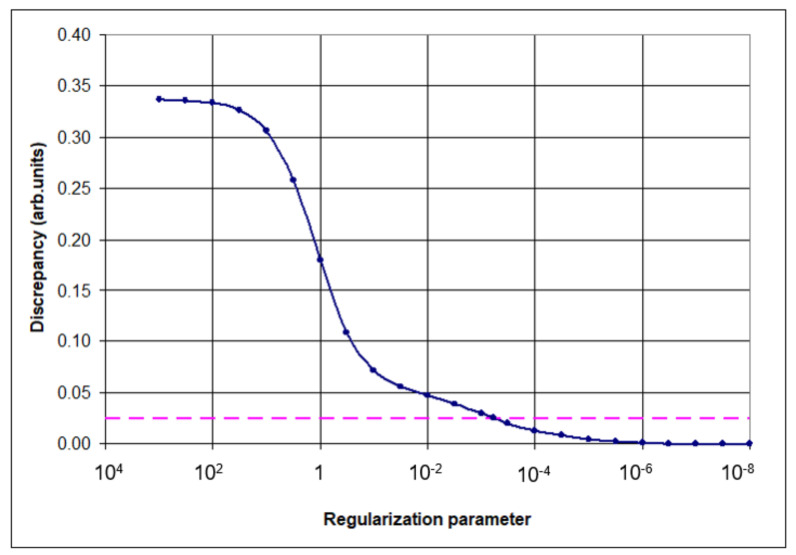
Dependence of the discrepancy between the experimental and calculated frequencies on the value of the regularization parameter α in the process of optimization. The choice of the regularization parameter in the optimization procedure is made according to the discrepancy principle [1,5] (dotted line).

For the complete automation of the procedure, no special constraints on matrix *B* were introduced, and the regularization parameter was chosen based on the desired approximation of the experimental frequencies within their given error level. The regularizing procedure is organized in a way that allows obtaining a complete and stable set of scale factors even in cases when some fundamental frequencies remain unknown. Figure 1 shows how the discrepancy between the observed and calculated at each stage of optimization frequencies depends on the value of the regularization parameter α during the optimization procedure. Obviously, the discrepancy decreases with decreasing regularization parameter; the dashed line shows the required error level, which in this case corresponds to *α* = 5.83∙10^−4^.

**Figure 2:**
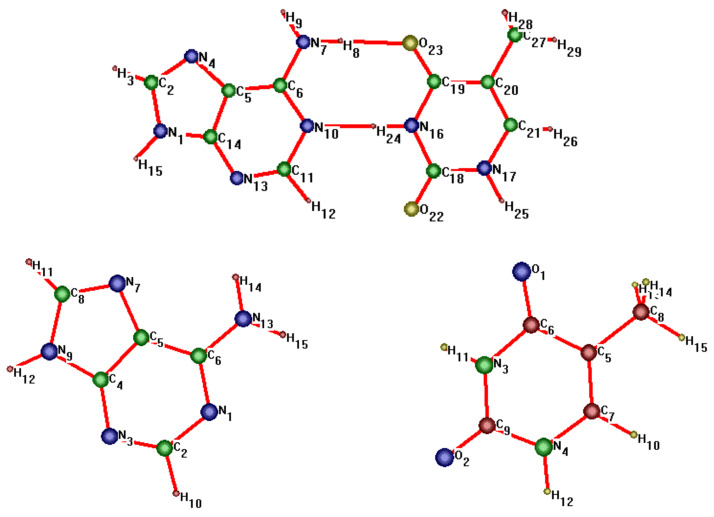
Adenine–thymine pair (**top**) and separate molecules (**bottom**).

**Figure 3:**
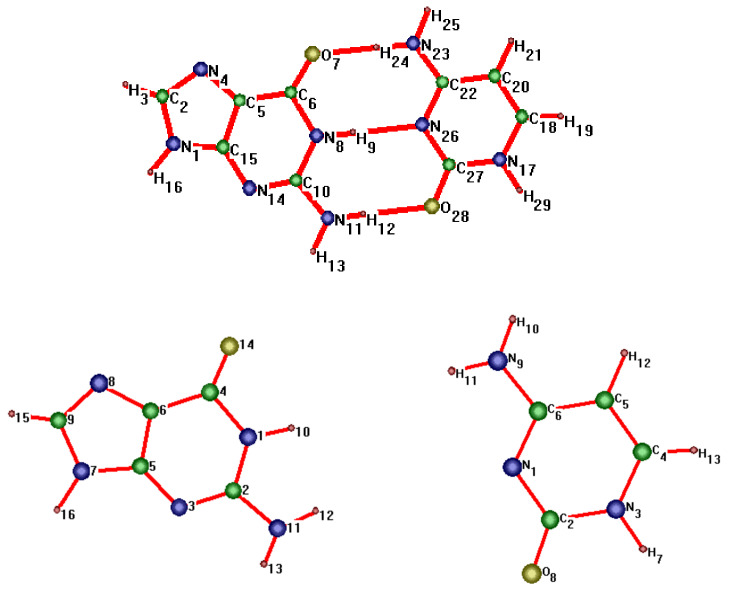
Cytosine-guanine pair (**top**) and separate molecules (**bottom**).

In this paper, we consider two DNA canonic pairs: adenine–thymine and guanine–cytosine. Figure 2 and Figure 3 show the pairs and separate DNA molecules.

In Table 3, we present the matrix of scale factors for adenine obtained on the basis of experimental frequencies. Atomic numbering is shown in Figure 2.

As it could be expected, most of the off-diagonal matrix *B* elements are small, and diagonal elements exhibit small deviations from unity. A similar structure of the scale matrix is observed in the case of other DNA bases; matrices are presented in Supplementary Materials—Tables S1–S4.

Vibrational frequencies of adenine calculated at the B3LYP/6-31G* level differ from the experimental one by 64 cm^−1^, while rms error in frequencies for the scaled force field is equal to 8.6 cm^−1^. For guanine, the B3LYP/6-31G* calculation results in the rms value of frequencies error equal to ~ 70 cm^−1^, while the scaled force field reduces this error to 5.7 cm^−1^. The experimentally observed vibrational frequencies together with their tentative assignments, as well as the frequencies calculated on B3LYP/6-31G* level and the frequencies obtained after the scaling procedure for all four DNA bases, can be found in Supplementary Materials—Tables S5–S8.

#### 2.3.4. Quantum Mechanical Calculations and Scaling of DNA Bases Canonic Pairs Hessians

Geometries and force fields for both pairs were calculated at the B3LYP/6-31G* level to ensure consistency of the scale factors with the individual DNA base calculations. For the scaling procedure, the scale matrix *B* for a DNA pair was built from the elements of the individual matrices for the bases, with the corresponding reordering of the matrix elements according to the order of atoms in the corresponding base pair. The off-diagonal elements of the DNA pair scale matrix corresponding to the interaction of atoms of different bases were taken to be zero. The “B3LYP/Scaled” column in Table 4 compares the theoretical B3LYP/6-31G* frequencies of adenine-thymine pare and corrected by the scale factors obtained for the individual molecules in Section 2.3.3.

Table 5 presents the results of a similar calculation for the guanine–cytosine pair. These frequencies may be subsequently used in analyzing experimental spectra.

## 3. Discussion

The results of using the scaling procedure directly in Cartesian coordinates for correcting the quantum mechanical Hessians of adenine and thymine (Table 4 and Table 5) demonstrate a satisfactory agreement between the experimental and fitted frequencies, which is consistent with the results obtained using the conventional Pulay scaling in internal coordinates. This allows us to conclude that the model for correcting the theoretical vibrational frequencies by scaling Cartesian force matrices appears reasonable.

Earlier, very close values of the diagonal scale factors were obtained for similar atoms in indole and pyrrole molecules [17]. The same is true for pairs of bonded atoms (C, H); this shows the good possibilities of transferring scale factors for atoms in a similar environment. The results of our calculations show that the optimized values of the scale factors *β_ij_* are the same for all pairs of atoms which are transferred to each other by symmetry operations for a particular molecule. Note that it is also possible to apply the procedure for each symmetry block individually, which gives a somewhat better frequency fit, similar to what is often done for the standard scaling approach.

A new numerical algorithm for calculating the scale factors for molecular force fields expressed in Cartesian coordinates reduces the difficulties associated with the choice of internal coordinates in complex molecules. The suggested method appears beneficial for calculating the vibrational spectra of large biological molecules, associates, polymers, and nanostructures in which the number of atoms exceeds hundreds and thousands, while only moderately accurate quantum chemistry methods may be applied. The results of such applications will be presented in future publications.

## 4. Conclusions

(a) An approach based on calculating the scale factor sets directly in Cartesian coordinates [16,17] is proposed to adjust the theoretical vibrational spectra of molecular associates with non-covalent interactions.
(b) Sets of scale factors in Cartesian coordinates (for the B3LYP/6-31G* level of theory) were calculated for four DNA bases within the model of correcting factor matrices based on the results of NBO analysis.
(c) Optimized (regularized) sets of scale factors (for B3LYP/6-31G * level) of adenine, thymine, cytosine, and guanine ensure the reproduction of experimental frequencies within the specified error.
(d) Calculated (regularized) sets of scale factors of DNA bases are applied for the “synthesis” (assembly) of correcting matrices of the corresponding DNA base pairs.
(e) Fitting theoretical frequencies by correcting molecular force fields directly in Cartesian coordinates significantly reduces the difficulties related to the choice of internal coordinates in bulky, complex molecules. Such modeling is practically useful in the case of large biological molecules, associates, polymers, etc.

## Figures and Tables

**Table 1:** Second-order perturbation theory analysis in NBO basis for non-bonded interactions in adenine and thymine molecules at the B3LYP/6-31G* level of theory.

Adenine			Adenine–Thymine		
Donor (i)	Acceptor (j)	E2	Donor (i)	Acceptor (j)	E2
LP N1	σ*(C2-N3)	12.61	LPN10	σ*(C11-N13)	10.89
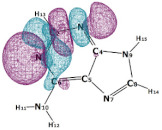			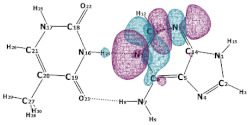		
LPN10	σ*(N1-C6)	50.42	LPN7	σ*(N10-C19)	21.59
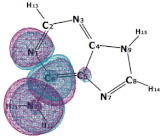			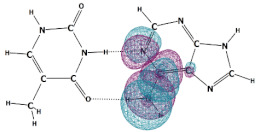		
Thymine			Adenine–Thymine		
LP(2)O2	σ*(N4-C9)	27.59	LP(2)O22)	σ*(N16-C18)	25.43
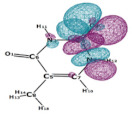			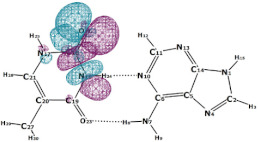		

**Table 2:** Second-order perturbation theory analysis in NBO basis for non-bonded interactions in guanine and cytosine molecules at the B3LYP/6-31G* level of theory.

Guanine and Cytosine			Guanine–Cytosine		
Donor (i)	Acceptor (j)	E2 ^a^	Donor (i)	Acceptor (j)	E2
LP(2)O14	σ*(C4-N1)	33.18	LPO7	σ*(C6-N8)	16.94
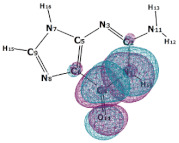			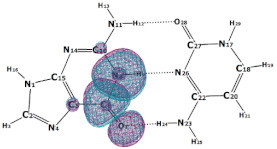		
LP(2)O14	σ*(C4-N1)	12.61	LP(2)O7	σ*(C6 -N8)	16.94
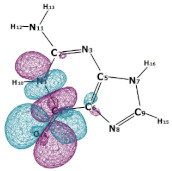			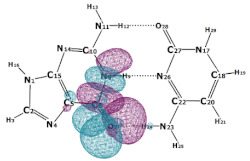		
LPN9	σ*(N1-C6)	47.18	LPN23	σ*(C22-N26)	61.91
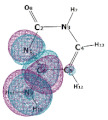			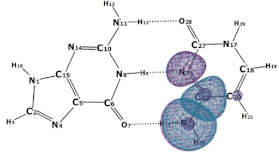		

^a^ E2—energy of hyperconjugative interactions.

**Table 3:** B3LYP/6-31G* Cartesian scale factors (matrix *B*) for adenine.

N1	0.992														
C2	0.003	1.015													
N3	0	−0.011	0.97												
C4	−0.011	−0.002	0.006	0.991											
C5	−0.007	−0.009	0.002	−0.002	1.007										
C6	−0.002	−0.006	0.009	0.002	0.002	0.946									
N7	−0.002	0.001	−0.005	0.005	−0.002	0.008	0.952								
C8	0.007	−0.001	0	0.005	−0.002	−0.007	0	1.007							
N9	−0.010	−0.001	0.005	0	−0.006	−0.008	−0.002	0.002	0.985						
N10	0.008	0.002	0.007	0.007	0.003	0.012	0.01	0.015	0.003	0.909					
H11	0.005	−0.001	0.008	−0.005	0.006	0	0.01	−0.013	0.003	0.008	0.985				
H12	0.007	0	0.005	−0.004	−0.002	0.008	0.013	0.003	0.002	0.012	−0.025	0.954			
H13	0.004	0	−0.008	0.01	0.001	0.016	0.011	−0.004	0.009	−0.002	0.003	0.005	0.947		
H14	−0.001	0.006	0.006	−0.003	0	0.013	−0.001	−0.001	0.006	0.006	0.013	0.014	0.002	0.93	
H15	0.003	0.001	0.004	−0.001	0.008	0.005	−0.001	−0.012	0.009	−0.002	0.001	0.007	0.002	0.009	0.966
	N1	C2	N3	C4	C5	C6	N7	C8	N9	N10	H11	H12	H13	H14	H15

**Table 4:** Adenine–thymine pair: theoretical B3LYP/6-31G* frequencies and frequencies calculated with the scaled force matrix (cm^−1^).

No	B3LYP/Scaled		No	B3LYP/Scaled		No	B3LYP/Scaled		No	B3LYP/Scaled	
1	3686	3489	22	1509	1459	43	996	976	64	534	514
2	3650	3443	23	1501	1456	44	991	973	65	520	501
3	3638	3430	24	1457	1428	45	946	929	66	472	468
4	3417	3331	25	1450	1423	46	912	897	67	409	388
5	3267	3097	26	1436	1389	47	912	886	68	408	383
6	3219	3046	27	1432	1385	48	839	823	69	391	363
7	3203	3043	28	1387	1368	49	810	808	70	314	298
8	3130	3001	29	1386	1343	50	803	797	71	303	294
9	3109	2997	30	1371	1334	51	766	784	72	299	286
10	3053	2967	31	1355	1297	52	751	773	73	288	277
11	3039	2927	32	1277	1259	53	745	747	74	228	227
12	1833	1776	33	1271	1244	54	741	730	75	171	179
13	1753	1700	34	1247	1225	55	730	702	76	166	158
14	1713	1670	35	1235	1197	56	688	655	77	150	147
15	1710	1635	36	1184	1162	57	668	646	78	116	111
16	1653	1626	37	1160	1131	58	637	618	79	111	111
17	1639	1606	38	1091	1058	59	615	604	80	103	101
18	1554	1533	39	1082	1051	60	582	568	81	67	65
19	1540	1497	40	1049	1022	61	574	554	82	63	61
20	1529	1493	41	1034	1015	62	553	527	83	34	32
21	1526	1474	42	1004	986	63	537	523	84	20	20

**Table 5:** Guanine–cytosine pair: B3LYP/6-31G* frequencies and frequencies for the scaled force matrix (cm^−1^).

No	B3LYP/Scaled		No	B3LYP/Scaled		No	B3LYP/Scaled		No	B3LYP/Scaled	
1	3692	3563	22	1460	1425	43	862	880	64	446	422
2	3674	3540	23	1453	1415	44	837	872	65	414	382
3	3650	3513	24	1428	1393	45	806	803	66	396	375
4	3623	3471	25	1395	1362	46	787	796	67	389	367
5	3405	3447	26	1395	1351	47	774	789	68	378	352
6	3264	3351	27	1368	1315	48	770	756	69	346	333
7	3248	3299	28	1326	1280	49	763	747	70	343	328
8	3244	3196	29	1313	1252	50	733	712	71	214	235
9	3226	2903	30	1233	1224	51	724	701	72	198	208
10	3175	2855	31	1195	1193	52	705	683	73	178	199
11	1784	1687	32	1178	1180	53	691	674	74	160	167
12	1756	1647	33	1148	1115	54	663	640	75	138	140
13	1732	1618	34	1129	1109	55	648	621	76	135	137
14	1714	1595	35	1121	1098	56	644	616	77	131	106
15	1690	1570	36	1078	1059	57	611	614	78	95	87
16	1670	1564	37	1065	1044	58	592	576	79	68	62
17	1629	1556	38	1005	1017	59	562	527	80	34	27
18	1577	1529	39	969	972	60	549	509	81	20	18
19	1567	1497	40	958	964	61	539	506			
20	1549	1467	41	946	937	62	517	473			
21	1540	1459	42	915	926	63	499	455			

## Data Availability

Data is contained within the supplementary materials available online.

## Supplementary Materials

The following supporting information can be downloaded online, Tables S1–S4: B3LYP/6-31G* Cartesian scale factors (matrix *B*) for adenine, thymine, guanine, cytosine respectively; Tables S5–S8: Quantum chemical (B3LYP/6-31G*), observed and scaled frequencies of adenine, guanine, thymine and cytosine respectively.

## Sample Availability

Samples of the compounds are not available from the authors.
