# Supplementary material for: Scaled in Cartesian Coordinates Ab Initio Molecular Force Fields of DNA Bases: Application to Canonical Pairs
Source: Molecules. 2022 Jan 10;27(2):427. doi: 10.3390/molecules27020427 (PMC8779963; doi:10.3390/molecules27020427)
Supplement: Supplementary file 1 [file molecules-27-00427-s001.zip › molecules-1536105-supplementary.pdf]

molecules

MDPI

Supplementary Materials

Scaled in Cartesian coordinates ab initio molecular force fields of DNA bases: application to canonical pairs

Igor Kochikov, Anna Stepanova and Gulnara Kuramshina

Table S1. B3LYP/6-31G* Cartesian scale factors (matrix *B*) for adenine.

N1	0.992														
C2	0.003	1.015													
N3	0.000	-0.011	0.970												
C4	-0.011	-0.002	0.006	0.991											
C5	-0.007	-0.009	0.002	-0.002	1.007										
C6	-0.002	-0.006	0.009	0.002	0.002	0.946									
N7	-0.002	0.001	-0.005	0.005	-0.002	0.008	0.952								
C8	0.007	-0.001	0.000	0.005	-0.002	-0.007	0.000	1.007							
N9	-0.010	-0.001	0.005	0.000	-0.006	-0.008	-0.002	0.002	0.985						
N10	0.008	0.002	0.007	0.007	0.003	0.012	0.010	0.015	0.003	0.909					
H11	0.005	-0.001	0.008	-0.005	0.006	0.000	0.010	-0.013	0.003	0.008	0.985				
H12	0.007	0.000	0.005	-0.004	-0.002	0.008	0.013	0.003	0.002	0.012	-0.025	0.954			
H13	0.004	0.000	-0.008	0.010	0.001	0.016	0.011	-0.004	0.009	-0.002	0.003	0.005	0.947		
H14	-0.001	0.006	0.006	-0.003	0.000	0.013	-0.001	-0.001	0.006	0.006	0.013	0.014	0.002	0.930	
H15	0.003	0.001	0.004	-0.001	0.008	0.005	-0.001	-0.012	0.009	-0.002	0.001	0.007	0.002	0.009	0.966
	N1	C2	N3	C4	C5	C6	N7	C8	N9	N10	H11	H12	H13	H14	H15

Table S2. B3LYP/6-31G* scale factors for thymine.

O1	0.926														
O2	0.014	0.917													
N3	-0.005	-0.003	1.122												
N4	-0.003	-0.005	-0.023	1.009											
C5	-0.002	0.000	-0.017	-0.017	0.963										
C6	0.033	0.008	-0.009	-0.003	-0.012	0.994									
C7	-0.003	0.007	-0.013	0.006	0.020	0.002	1.019								
C8	0.004	0.001	-0.015	-0.007	-0.004	-0.014	-0.002	0.975							
C9	0.002	0.029	-0.006	-0.010	0.009	-0.009	-0.004	-0.002	1.005						
H10	0.014	0.016	0.003	-0.013	-0.009	0.015	0.009	0.009	0.003	0.964					
H11	-0.010	-0.011	-0.001	0.036	0.013	0.013	-0.017	0.016	-0.001	-0.022	0.942				
H12	0.016	0.005	-0.006	0.028	0.033	-0.029	-0.033	0.005	-0.022	0.011	0.028	0.955			
H13	0.004	0.006	-0.009	0.001	0.010	0.001	0.001	0.010	0.001	0.000	0.004	0.004	0.975		
H14	0.004	0.006	-0.009	0.002	0.010	0.001	0.001	0.010	0.001	0.000	0.004	0.004	-0.007	0.975	
H15	0.005	0.008	-0.010	-0.002	0.002	0.007	0.007	0.011	0.001	-0.001	0.005	0.000	-0.002	-0.002	0.970
	O1	O2	N3	N4	C5	C6	C7	C8	C9	H10	H11	H12	H13	H14	H15

Table S3. B3LYP/6-31G* scale factors for guanine.

N1	1.020															
C2	-0.006	0.841														
N3	-0.009	0.002	1.018													
C4	-0.005	0.009	0.014	0.953												
C5	0.006	0.015	0.017	-0.010	0.999											
C6	0.001	0.044	-0.006	0.004	0.003	0.938										
N7	0.001	0.021	-0.018	-0.007	0.003	-0.018	1.039									
N8	0.003	0.028	-0.005	-0.011	-0.010	-0.035	-0.008	0.976								
C9	0.001	0.031	-0.009	-0.003	0.003	0.009	-0.016	-0.016	0.989							
H10	0.014	-0.011	0.025	0.003	-0.003	0.003	-0.007	0.008	-0.017	0.924						
N11	-0.020	-0.023	-0.022	-0.013	0.006	0.020	0.004	0.043	0.027	0.004	0.934					
H12	-0.013	-0.010	-0.002	-0.001	0.002	0.003	0.009	0.013	0.022	0.011	0.008	0.973				
H13	0.005	0.006	-0.009	0.023	-0.009	0.010	0.000	0.011	0.012	-0.008	0.008	-0.022	0.970			
O14	0.002	0.016	0.002	0.042	0.007	0.010	-0.008	-0.004	-0.001	0.002	0.007	-0.003	-0.003	0.938		
H15	-0.021	0.020	0.007	-0.011	-0.006	0.027	-0.012	-0.035	-0.008	0.051	0.010	-0.001	0.001	-0.003	0.981	
H16	0.019	0.014	-0.008	0.008	-0.025	-0.015	0.015	0.039	-0.024	-0.001	0.005	0.009	0.002	-0.005	-0.001	0.967
	N1	C2	N3	C4	C5	C6	N7	N8	C9	H10	N11	H12	H13	O14	H15	H16

Table S4. B3LYP/6-31G* scale factors for thymine.

N1	0.917												
C2	0.013	0.937											
N3	-0.028	-0.006	1.046										
C4	0.005	-0.014	0.000	0.994									
C5	-0.004	0.013	0.000	0.024	0.968								
C6	0.025	0.021	0.027	-0.005	0.025	0.888							
H7	-0.003	-0.004	0.010	-0.007	0.007	-0.020	0.988						
O8	0.011	0.045	-0.012	-0.007	0.001	0.017	0.001	0.925					
N9	0.005	0.008	-0.004	-0.004	-0.008	0.014	0.017	0.007	0.957				
H10	0.020	-0.009	0.029	-0.008	-0.005	0.016	0.000	0.016	0.019	0.920			
H11	0.017	0.008	-0.020	0.009	0.000	-0.001	0.010	0.013	0.013	-0.005	0.954		
H12	-0.003	-0.004	-0.021	-0.023	-0.001	-0.013	-0.003	-0.013	-0.011	0.004	-0.010	1.104	
H13	0.022	-0.010	-0.025	0.033	-0.022	0.004	0.000	-0.008	-0.015	0.000	0.008	-0.008	1.019
	N1	C2	N3	C4	C5	C6	H7	O8	N9	H10	H11	H12	H13

Table S5. Quantum chemical (B3LYP/6-31G*), observed and scaled frequencies of adenine

No	Tentative assignment	Hirakawa et al. [1]		Novak et al. [2]	B3LYP/6-31G*	
		IR	Raman	IR (Ar matrix)	Calc.	Scaled
1	NH_2 as str			3565	3720	3565
2	N9-H str			3498	3651	3499
3	NH_2 sym str			3441	3598	3448
4	C8-H str			3057	3266	3059
5	C2-H str			3041	3190	3041
6	R6 str , NH_2 sc	1673 vs	1677vw	1633	1681	1635
7	R6 str	1637w	1612	1612	1652	1614
8	NH_2 sc , R6 str	1604vs	1508	1590	1628	1598
9	R6 str,C2Hin-pl-bend	1508	1482	1482	1534	1484
10	R5 str	1451	1462-42	1474	1522	1475
11	R5 str	1421	1419	1419	1447	1419
12	C2H in-plane-bend	1368	1371	1389	1432	1390
13	R6 str	1335	1332	1345	1380	1335
14	C6N str	1309	1307	1328	1371	1328
15	R5 str	1253	1249	1290	1344	1292
16	C8H in-plane-bend	1234	1235	1240	1277	1238
17	NH_2 wag	1157	1163	1229	1258	1227
18	R5-in-plane-def	1126	1126		1152	1127
19	N9-H in-plane-bend	1025	1024	1127	1091	1060
20	NH_2 tw	951		1032	1029	1003
21	C2H-out-of-plane-end	940	952	1005	972	950
22	R5- in-plane-def	913	941	927	944	927
23	R6- in-plane-def	872	899	927	901	886
24	C8H-out-of-planebend	849		887	844	823
25	R6,R5 def (breath)	844	846	802	809	802
26	R5-o-o-pl-defingpuck)	797		717	728	700
27	C6N out-of-planebend	723	797	610	686	655
28	R5-in-plane-def (sqz)	684	724	591	670	647
29	R6-out-of-pl-def (tor)	660		583	620	603
30	R6-in-plane def	641	622	566	578	568
31	R6-in-plane-def	622	560	513	535	528
32	R5-out-of-pl-def (tor)	543	535		530	513
33	NH_2 rock	530	530		516	503
34	C6-N torsion	380	331	503	507	495
35	N9H out-of-pl bend	337			313	288
36	R6-R5–out-of-pl-dftor	249			299	280
37	R6-R5- out-of-pl-def		242	276	272	254
38	R6,R5out-ofpl-def btfl		238	242	214	213

39	R6,R5-out-of-pl-def tw				214		166	159

Table S6. Comparison of observed frequencies, theoretical B3LYP/6-31G* and scaled force field frequencies (in cm^{-1}) of guanine.

No	Tentative assignment	R.Lopes et al. [3]			B.Giese et al.[4]	PBEPBE/ DGDZVP	B3LYP/6-31G*	
		INS	Raman	IR	Raman (polycr)	Theor.	Theor.	Scaled
1	N-H str	1417	3348	3325		3577	3668	3507
2	N-H str		3164	3178		3541	3646	3506
3			3110	3113		3486	3586	3490
4				3064		3463	3561	3456
5		1406	2992	2989		3194	3269	3057
		1375	2898	2904				
				2846				
	CH str	1269	2708	2696				
6		1226		1697		1755	1833	1679
7		1190	1674	1672	1675	1631	1689	1606
8		1178			1602	1580	1633	1555
9	C=O str	1160	1549	1562	1575	1566	1619	1552
10	C=O str	1109	1549	1552	1545	1516	1570	1481
11	C=C str	1045			1503	1475	1526	1470
		946	1479	1475	1479			
		909	1466	1465	1468			
12		886	1421	1417	1421	1408	1449	1422
13		847	1390	1373	1390	1350	1398	1389
14		802	1359		1361	1341	1369	1361
15		786	1265	1261	1265	1293	1344	1267
16		737	1232	1215	1234	1265	1315	1234
17		705	1185	1173	1186	1138	1179	1188
18		694	1158		1155	1120	1153	1158
19		657	1121	1120		1047	1082	1050
20		601	1046	1043	1048?	1044	1079	1049
21		570				1016	1052	1047
22		562	935	949	937	910	947	936
		557						
23		551	878	881	879	809	836	877
24		546	848	850	848	769	813	840
25		540	803	791	775	740	773	766
		507	775	779				
26		499	727	727	710	715	736	704
27		403	711	702		675	703	685
28		379	692	688	693	646	670	682
29		361	650	644	649	638	664	649
30		333	601	604	603	618	629	602
31		238			562	604	605	558
32		196				569	598	540
33		177	540			506	531	495
34		158				500	526	486
35		138			495	471	486	410
36		127			397	346	363	355
37		124			340	324	341	334

38	90	357	320	333	328
39	60		306	317	299
40	30		193	203	198
41			150	162	156
42			132	140	142

Table S7. Comparison of theoretical B3LYP/6-31G* and observed frequencies (in cm^{-1}) of thymine.

No	Tentative assignment	Szczepaniak et al. [5]	Singh et al. [6]		Rastogi et al. [7]			B3LYP /6-31G*	Scaled
		IIR	IR in KBr	Raman polycr.	IR gas	Raman	Raman cr.		
1	N-H str	3470 IR	3185 vs	3185sh	3484			3641	3474
2	N-H str	3432 IR	3160 ch	3160sh	3437	3070		3603	3432
3	CH str	3078 Ra	3060 m	3060w	3076+	3044		3219	3080
4	CH3 str	2997 Ra	3005 w	3000w	2984	3021		3132	2999
5	CH3str	2992 IR			2941			3111	2992
6	CH3 str	2969				2968		3054	2968
		2939	2925s	2925m		2919			
			2895sh	2900w					
7	C=O str	1767			1772			1845	1771
8	C=O str	1711	1738vs	1740vs	1725	1720	1718	1793	1715
9	C=C str	1668	1680vs	1680s	1668	1670	1674	1717	1671
			1640sh	1645sh	1630m		1600		
			1505sh	1505w	1518				
10						1488	1492	1524	1512
11		1472	1465sh	1465 s	1463			1506	1472
12		1455	1445vs	1445vs			1437	1500	1453
13		1431		1430s		1432		1448	1431
14		1405	1420c	1420s	1409	1406	1413	1431	1405
		1388	1380s		1393				
15		1367	1360sh	1375vs		1367	1379	1411	1383
16		1357		1360sh			1373	1385	1366
			1300w	1300sh		1259			
17		1220	1240vs	1240vs		1245	1263	1238	1221
18		1183	1195vs	1200vs		1214	1252	1207	1183
19		1139	1145w	1150w	1178		1221	1162	1138
20		1046	1030s	1030vs	1031	1025	1161	1082	1045
21		1004	985s	995vs	963	983	1053	1031	1005
22		959	940s	940vs	931		986	969	959
23		889		900w	895		938	910	883
24			850vs	845s				806	802
25		799	815vs	815vs	804			767	790
26		763	760s	760vs	767		810	749	760
27		754	745s	740vs	755		747	738	746
28		727	710sh		689		624	690	705
29				640w	658		563	605	597
30			610mw	610m			474	562	546
31			560vs		541		434	545	500
32			462vs		462			460	457
33			420s					397	371
34							325	387	364

No									
35							292	298	294
36								276	268
37							175	153	161
38							157	136	133
39							121	112	109

Table S8. Comparison of observed frequencies, theoretical B3LYP/6-31G* and frequencies for the scaled force field (in cm^{-1}) of cytosine

No	Tentative assignment	Szczesniak [8]	Radchenko et al. [9]		Mathlouthi et al. [10]	Nowak et al. [11]		B3LYP 6-31G*	Scaled
		IR	IR solid	Raman polycr	Ne	Ne	Ar		
1	N-H str	3587			3618-3609	3618	3592	3711	3592
2	N-H str	3559			3575	3575	3564	3617	3563
3	CH str	3468			3527	3461	3446	3589	3446
4	CH3 str	3438			3501			3239	3312
5	CH3str				3474			3216	3173
					3461				
					3457				
6	CH3 str	1749	1705	1708				1820	1627
7		1729			1770	1625	1623	1708	1598
		1714			1760		1589		
8	C=O str	1670	1657	1666	1730-1725	1592	1600	1660	1570
9	C=O str	1665			1686	1576	1575	1582	1499
10	C=C str	1655	1616	1620	1678	1569	1570	1522	1436
		1620				1563	1561		
		1617		1582	1668				
11		1595			1659	1495	1496	1454	1381
		1587	1540	1542	1642	1482	1439		
		1559	1505	1502	1623	1441	1427		
12		1535	1469	1470	1602-1597	1430	1379	1367	1326
		1490			1592	1382			
13		1473			1576-1569-1563	1380		1263	1275
		1438	1366	1376	1554	1338	1333		
14		1426			1540	1324	1320	1224	1196
		1376			1495-1492	1258?	1257		
		1336	1280	1284	1475	1198	1196		
		1318	1240	1260	1441	1113	1110		
15					1430	1109	1108	1134	1111
		1256			1436	1085	1083		
		1223			1427	989	980		
16		1209	1155	1158	1423	982	855?	1105	1082
17		1193		1118	1397	948?	807	991	980
18			1100			848?	781	955	924
19		1123				809	751?	926	904
20		1107				796	710	771	804
21		1090	1012	1022		784?	601?	770	776
22		1082		1002		711	569	761	699
23				980		600?	520	732	699
24			955	904		525	557	630	581

25	980	866		553	498	577	556
26		822		511	441	549	524
27		794	798	498	451	534	510
28	817	783		494	343	526	491
29	807	758		443	350	393	428
30	780	700	710	342	297	359	339
31	767					328	295
32	749					204	193
33	709	602	608			138	135
	635		578				
	613		558				
	574		546				
	567						
	535						
	519		490				
	507		460				
	498		414				
	441						

1 Hirakawa, A. Y.; Okada, H.Y.; Sasagawa, S.; Tsuboi, M. Infrared and Raman spectra of adenine and its ^{15}N and ^{13}C substitution products. *Spectrochimica Acta A* **1985**, ***4l***, 209-216. https://doi.org/10.1016/0584-8539(85)80099-4

2 Nowak, M.J.; Lapinski, L.; Kwiatkowski, J.S.; Leszczynski, J. Molecular structure and infrared spectra of adenine. Experimental matrix isolation and density functional theory study of adenine ^{15}N isotopomers. *J. Phys. Chem.* **1996**, *100*, 3527-3534. https://doi.org/10.1021/jp9530008

3 Lopes, R.P.; Marques, M.P.M.; Valero, R.; Batista de Carvalho, L.A.E. A combined study using vibrational spectroscopy and theoretical methods. *J. of Spectroscopy* **2012**, *27*, 273–292. https://doi.org/10.1155/2012/168286

4 Giese, B.; McNaughton, D. Density functional theoretical (DFT) and surface-enhanced Raman spectroscopic study of guanine and its alkylated derivatives. Part 1. DFT calculations on neutral, protonated and deprotonated guanine. *Phys. Chem. Chem. Phys.* **2002**, *4*, 5161–5170. https://doi.org/10.1039/B203829C

5 Szczepaniak, K.; Szczesniak, M.; Person, W.B. Raman and infrared spectra of thymine. A matrix isolation and DFT study. *J. Phys. Chem. A* **2000**, *104*, 3852-3863. https://doi.org/10.1021/jp994410p

6 Singh, J.S. FTIR and Raman spectra and fundamental frequencies of biomolecule: 5-Methyluracil (thymine). *J. Mol. Struct.* **2008**, *876*, 127–133. https://doi.org/10.1016/j.molstruc.2007.06.014

7 Rastogi, V.K. ; Singh, C.; Jain, V.; Alcolea Palafox, M. FTIR and FT-Raman spectra of 5-methyluracil (thymine). *J. Raman Spectrosc.* **2000**, *31*, 1005–1012. 0.1002/1097-4555(200011)31:11<1005::AID-JRS636>3.0.CO;2-7

8 Szczesniak, M.; Szczepaniak, K.; Kwiatkowski, J. S.; KuBulat, K.; Person, W. B. Matrix isolation infrared studies of nucleic acid constituents. 5. Experimental matrix-isolation and theoretical ab iInitio SCF Molecular orbital studies of the infrared spectra of cytosine monomers. *J. Am. Chem. Soc.* **1988**, *110*(25), 8319-8330. https://doi.org/10.1021/ja00233a006

9 Radchenko, E.D. ; Sheina, G.G.; Smorgio, N.A.; Blagoi, Yu.P. Experimental and theoretical studies of molecular structure features of cytozine. *J. Mol. Struct.*, **1984**, *116*, 387-396.

10 Mathlouthi, M.; Seuvre, A.; Koenig, J. F.t.-i.r. and laser-Raman spectra of cytosine and cytidine. *Carbohydr. Res.* **1986**, *146* (1), 1-13. https://doi.org/10.1016/0008-6215(86)85019-4

11 Nowak, M. J.; Lapinsky, L.; Fulara, J. Matrix isolation studies of cytosine: The separation of the infrared spectra of cytosine tautomers. *Spectrochimica Acta A*, **1989**, *45*(2), 229. https://doi.org/10.1016/0584-8539(89)80129-1
